# ROS-Driven Oxidative Modification: Its Impact on Chloroplasts-Nucleus Communication

**DOI:** 10.3389/fpls.2019.01729

**Published:** 2020-01-23

**Authors:** Chanhong Kim

**Affiliations:** Shanghai Center for Plant Stress Biology and Center of Excellence in Molecular Plant Sciences, Chinese Academy of Sciences, Shanghai, China

**Keywords:** reactive oxygen species, retrograde signalling, chloroplast, photosystem, oxidative modification

## Abstract

As a light-harvesting organelle, the chloroplast inevitably produces a substantial amount of reactive oxygen species (ROS) primarily through the photosystems. These ROS, such as superoxide anion, hydrogen peroxide, hydroxyl radical, and singlet oxygen, are potent oxidizing agents, thereby damaging the photosynthetic apparatus. On the other hand, it became increasingly clear that ROS act as beneficial tools under photo-oxidative stress conditions by stimulating chloroplast-nucleus communication, a process called retrograde signaling (RS). These ROS-mediated RS cascades appear to participate in a broad spectrum of plant physiology, such as acclimation, resistance, programmed cell death (PCD), and growth. Recent reports imply that ROS-driven oxidation of RS-associated components is essential in sensing and responding to an increase in ROS contents. ROS appear to activate RS pathways *via* reversible or irreversible oxidation of sensor molecules. This review provides an overview of the emerging perspective on the topic of “oxidative modification-associated retrograde signaling.”

The post-translational modifications (PTMs) are essential in initiating intra- and inter-cellular signaling cascades. Such PTMs, including acetylation, phosphorylation/de-phosphorylation, ubiquitination, sumoylation, and redox-related protein modifications, are ubiquitous cellular events dynamically deployed in signaling network under various developmental and stress conditions (Schweppe et al., 2003; Apel and Hirt, 2004; Jensen, 2004; Buchanan and Balmer, 2005; Stulemeijer and Joosten, 2008). Unlike extraplastidic signaling cascades where the protein modifications have been extensively investigated, the current understanding of the potential impact of PTMs in triggering chloroplast-to-nucleus retrograde signaling pathways is mostly limited in the context of the reversible redox modifications (Tikkanen et al., 2012; Dietz et al., 2016). Even though chloroplasts are prime subcellular organelles producing reactive oxygen species (ROS), reactive electrophile species (RES) mainly through lipid peroxidation (Girotti and Kriska, 2004; Mueller et al., 2008), and stress hormones under both biotic and abiotic stress, the converging PTM events in stress-related proteins are mostly unexplored. In particular, oxidative PTMs by ROS and its interconnection with other PTMs, which may function in protein turnover, signaling, and metabolism, are poorly understood. Given that chloroplasts produce harmful reactive species, in this review paper, the current understanding of oxidative PTMs and its associated retrograde signaling pathways are mainly discussed, which may provide a new prospect in the field of chloroplast-to-nucleus retrograde signaling research.

## Two Singlet Oxygen Sensors Undergo Oxidative Modification

The first breakthrough which has changed the classical view of ROS from toxic to signaling-associated agents arose from the identification of *fluorescent* (*flu*) mutant of *Arabidopsis thaliana* (Meskauskiene et al., 2001). Given that the FLU protein negatively regulates the Mg-branch of tetrapyrrole synthesis, overaccumulation of protochlorophyllide (Pchlide, end product in the dark) in the absence of light is inevitable in the *flu* mutant plants. The increase of free Pchlide, a photosensitizer, results in the generation of singlet oxygen (^1^O_2_) upon illumination, leading to rapid cell death in young seedlings and growth inhibition in mature plants (op den Camp et al., 2003). ^1^O_2_-associated global transcriptome analyses reveal that a substantial number of nuclear genes (mostly related to stress responses) become upregulated upon ^1^O_2_ release in chloroplasts. The gene expression changes precede the onset of cell death (op den Camp et al., 2003; Kim et al., 2012). At first glance, the rapid expression of nuclear-encoded stress-related genes was thought of as a consequence of ^1^O_2_ cytotoxicity. However, the subsequent forward genetic screen has provided a clue that the ^1^O_2_-associated transcriptome, as well as the cell death in young seedlings (or grown inhibition in mature plants) are likely to be mediated *via* activation of chloroplast-to-nucleus retrograde signaling (hereafter RS) (Wagner et al., 2004; Kim et al., 2008). The nuclear-encoded plastid protein EXECUTER (EX) 1 and its close homolog EX2 appear to mediate the stress responses in both young seedlings and mature plants of *flu* (Lee et al., 2007). Therefore, the genetic inactivation of EX1 and EX2 nearly abrogates ^1^O_2_-triggered stress responses, including nuclear gene expression changes (Lee et al., 2007). This indicates that chloroplasts lacking both EX1 and EX2 become insensitive to ^1^O_2_ generated in the *flu* mutant plants upon a dark-to-light shift. While EX1 plays a significant role in ^1^O_2_ signaling, EX2 acts as a modulator. The loss of EX2 in *flu* only alters the ^1^O_2_-associated nuclear transcriptome, but neither cell death nor growth inhibition (Kim et al., 2012). Besides, the absence of developmental defects in *ex1ex2* mutant plants (Kim et al., 2012) suggests that EXs are essentially functioning under photo-oxidative stress conditions in which chloroplasts enhance the level of ^1^O_2_.

**Figure 1:**
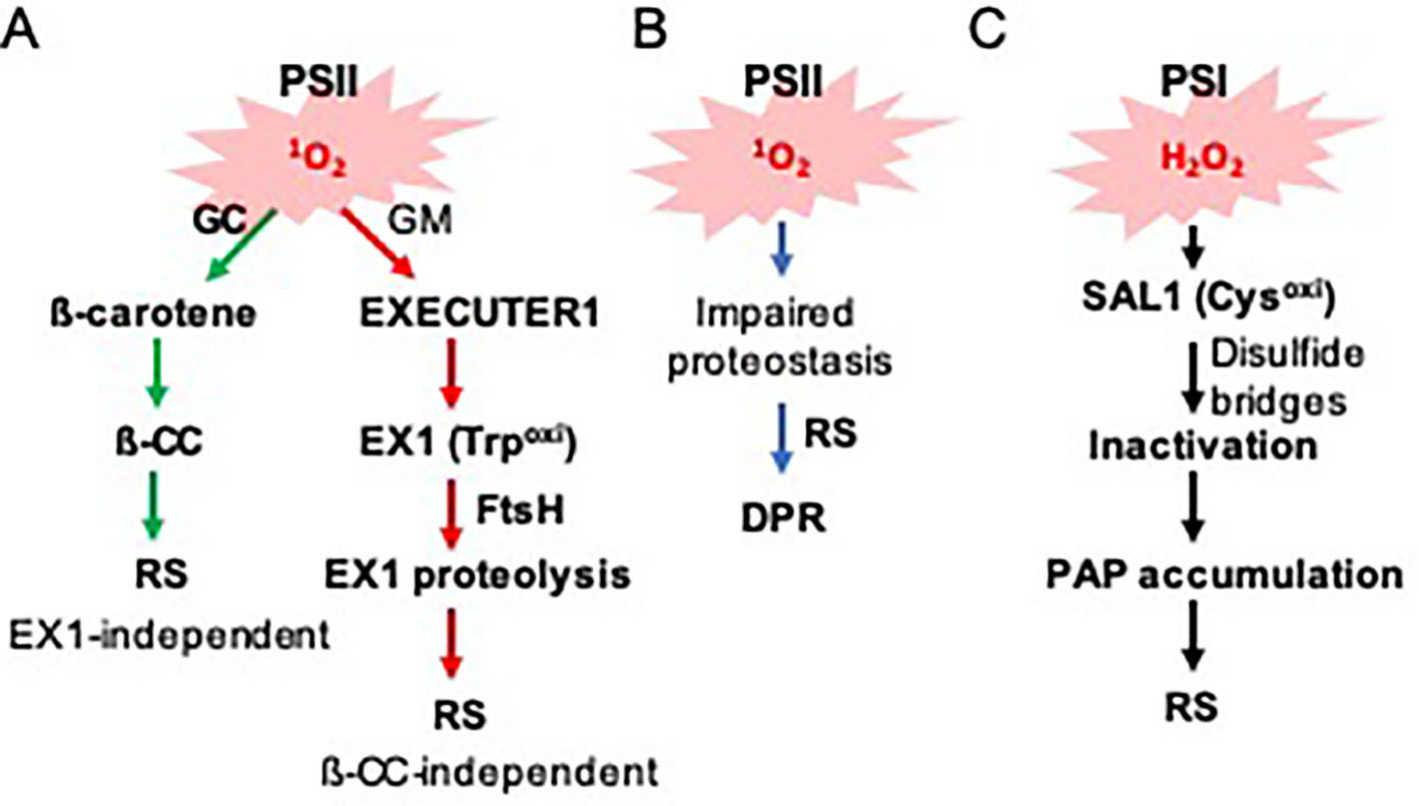
Photosystems-driven ROS trigger distinct RS pathways. **(A)** PSII-associated ^1^O_2_ sensors beta-carotene and EX1 mediate RS in the grana core (GC) and grana margins (GMs), respectively. ^1^O_2_ oxidizes ß-carotene and EX1, resulting in the generation of ß-CC and EX1(Trp643^oxi^). While ß-CC serves as a volatile RS molecule, it is unclear how EX1(Trp643^oxi^) degradation alters the expression of ^1^O_2_-responsive nuclear genes. **(B)** ^1^O_2_ constantly damages PSII core proteins, which leads to the accumulation of damaged proteins in the chloroplasts of the *var2* mutant. The impaired proteostasis triggers a DPR *via* an EX1-independent pathway. **(C)** PSI-driven ROS (such as H_2_O_2_) inactivate the SAL1 enzyme *via* intra- and/or inter-disulfide bond formation, leading to the accumulation of its substrate PAP. PAP then mediates RS.

Mostly based on the genetic and transcriptome studies, EX1 has long been considered as a putative ^1^O_2_ sensor (**Figure 1A**). The first mechanistic insight on how EX1 mediates ^1^O_2_ signaling was only recently attained (Wang et al., 2016; Dogra et al., 2019b). Interestingly, membrane-bound FtsH2 protease participates in ^1^O_2_ signaling by promoting EX1 degradation upon release of ^1^O_2_ (Wang et al., 2016). FtsH2 is a major subunit of the hetero-hexameric FtsH complex, which functions in the proteolysis of damaged photosystem II (PSII) reaction center (RC) proteins. Inactivation of FtsH2 thus substantially attenuates ^1^O_2_ signaling and its related stress responses in *flu*, like in the *flu ex1* mutant (Dogra et al., 2017). This finding also suggested that EX1 might undergo post-translational modification, thereby promoting EX1 degradation. Consistent with the notion, it appears that the tryptophan-643 residue (Trp643) undergoes ^1^O_2_-dependent oxidation (Dogra et al., 2019b). Oxidation of the indole side chain of Trp leads to the production of different Trp variants, such as keto-amino-hydroxy derivative oxindolylalanine (Oia), dihydro-hydroxy derivative N-formylkynurenine (NFK), and kynurenine with the corresponding mass shifts of + 16, + 32, and + 4 Da, respectively (Anderson et al., 2002; Dogra et al., 2019b). A substitution of Trp643 with ^1^O_2_-insensitive amino acid residues, such as Alanine and Leucine, completely inactivates EX1. However, the modified EX1 proteins appear to be unexpectedly unstable and fail to accumulate despite the abundance of the cognate mRNA transcripts. Presumably, they failed to localize in the grana margin or become misfolded, facilitating their degradation. Because of this instability, it is unclear which of the assumptions, or even both, are responsible for the impaired ^1^O_2_ signaling. However, this finding suggests that the accumulation of EX1 in the grana margins, wherein it associates with various proteins, and its FtsH-dependent proteolysis upon ^1^O_2_ burst, are pivotal in initiating ^1^O_2_ signaling (**Figure 1A**).

The deletion of DUF3506 (the domain of unknown function 3506; now dubbed singlet oxygen sensor [SOS] domain) containing Trp643 also impairs ^1^O_2_ signaling with remaining EX1 stability in response to ^1^O_2_. In 2004, Klaus Apel and his coworkers reported two independent *ex1* alleles, each of which contains a missense mutation in the SOS domain resulting in Phenylalanine-528-Cysteine and Glycine-646-Aspartic acid, respectively. It turned out that these mutated EX1 proteins cannot undergo Trp643 oxidation, underpinning the essential role of Trp643 in sensing ^1^O_2_ and initiating cognate signaling (Dogra et al., 2019b). In fact, earlier studies found that amongst the ^1^O_2_-sensitive amino acid residues, only Trp can scavenge ^1^O_2_ *via* both physical quenching and chemical reactions. Thus, Trp is highly susceptible and reacts rapidly with ^1^O_2_ (Davies, 2003; Pattison et al., 2012).

Another ^1^O_2_ sensor, ß-carotene, also undergoes oxidation *via* ^1^O_2_ under light stress conditions (**Figure 1A**). EX1 mediates ^1^O_2_ signaling in the grana margin (non-appressed), while ß-carotene functions in the grana core (appressed) (Dogra et al., 2018). These two signaling cascades are distinct, as evident in the substantial difference of downstream target genes (Dogra et al., 2017; Dogra et al., 2018). Excess light-generated ^1^O_2_ oxidizes ß-carotene, which leads to the production of its volatile compounds such as ß-cyclocitral (ß-CC) which belongs to the RES. ß-CC then induces numerous nuclear-encoded genes independently of EX1 and EX2 (Ramel et al., 2012; Ramel et al., 2013b; Ramel et al., 2013a). Given that excess light results in the generation of ß-CC in PSII RC where ß-carotene acts as a ^1^O_2_ scavenger, it is tempting to suggest that there might be a certain threshold level of ß-CC required for initiating RS. While the source of ^1^O_2_ for ß-carotene oxidation is evident, the source of EX1 oxidation is yet unclear. Considering the exclusive localization of EX1 in the grana margin and the extremely short lifespan of ^1^O_2_ (Apel and Hirt, 2004; Asada, 2006), it is difficult to suggest that ^1^O_2_ generated in PSII in the grana core moves to the grana margin wherein EX1 senses it. Perhaps, as suggested recently (Dogra et al., 2018), the impaired reassembly process of PSII in the grana margin may cause the generation of ^1^O_2_ under photo-oxidative stress conditions. In the grana margin, EX1 is associated with PSII RC proteins, FtsH protease, chlorophyll synthesis enzymes, and proteins of the chloroplast translation apparatus (Wang et al., 2016). These proteins are all implicated in PSII reassembly. Since PSII reassembly requires newly synthesized chlorophyll molecules, it is possible that, by any chance, free chlorophyll or its precursors released under stress generate ^1^O_2_. EX1 may sense such ^1^O_2_ generated in the grana margin. However, at present, we cannot exclude a possibility that any oxidized products (including RES) by ^1^O_2_ oxidizes EX1 protein (Dogra et al., 2018; Dogra et al., 2019b). Therefore, finding the source of EX1 oxidation would shed light on the underlying mechanism of EX1 oxidation further.

## Damaged/Unfolded/Misfolded Protein Responses *via* RS

^1^O_2_ is a prime ROS causing photodamage in PSII RC proteins (Apel and Hirt, 2004; Yamamoto et al., 2013). Especially the D1 protein has been implicated as a major target of ^1^O_2_ because of its proximity to the chlorophyll a molecule (called P680) in PSII RC. It is known that the excited triplet state of P680 serves as a photosensitizer. Earlier studies demonstrated that high light stress results in Trp oxidation in the D1 protein, not in all but certain Trp residues (Dreaden et al., 2011; Dreaden Kasson et al., 2012). Dogra et al., (2019b) also confirmed that these Trp residues in D1 undergo oxidation, resulting in the generation of oxidized Trp variants, including NFK. Nonetheless, photodamage in PSII RC proteins facilitates a process called PSII repair, which involves a series of steps: 1) migration of damaged PSII RC from the grana core to the grana margin; 2) degradation *via* the FtsH protease; 3) reassembly *via* protein *de novo* synthesis; and 4) re-migration from the grana margin to the grana core (Kato and Sakamoto, 2018). The hetero-hexameric FtsH protease comprises four subunits, FtsH1, 2, 5, and 8. Among them, FtsH2 and FtsH5 are pivotal for establishing a functional FtsH protease, as evident in the emergence of leaf variegation in the cognate mutant plants (Yu et al., 2005). Besides, the loss of FtsH2 results in the accumulation of damaged PSII proteins relative to wild-type plants and in the failure of survival under very mild light stress conditions wherein wild-type plants can rapidly acclimate (Duan et al., 2019).

Not only PSII proteins but also other chloroplast proteins appear to accumulate in the chloroplasts of *var2* mutant lacking FtsH2. Interestingly, a substantial number of proteins, which are known to function in protein quality control (PQC), are accumulating in *var2* (Dogra et al., 2019a). These proteins include heat shock proteins and heat shock transcription factors as well as a suite of proteases (such as the ATP-dependent caseinolytic protease Clp). Besides, detoxification- and redox-related proteins were found to accumulate in *var2*, coinciding with an increase in ROS in the chloroplasts (Kato et al., 2009). The resulting global transcriptome analysis of *var2* versus wild-type plants revealed that the accumulation of PQC-related proteins seems to be transcriptionally controlled *via* RS (Dogra et al., 2019a). This molecular phenotype (upregulation of a suite of proteins involved in PQC) resembles one so-called unfolded/misfolded protein response, namely UPR (Walter and Ron, 2011). Considering the plausible interrelation between the accumulation of damaged chloroplast proteins and the induction of PQC/detoxification/redox-related nuclear genes, we dubbed this UPR-like response as a damaged protein response (DPR) (**Figure 1B**).

The UPR was first characterized in the endoplasmic reticulum (ER) and is an ubiquitously conserved cellular system eliminating misfolded/unfolded proteins, thereby maintaining proteostasis (Kozutsumi et al., 1988). A presence of chloroplast-mediated UPR (cpUPR) was first reported in the green unicellular alga *Chlamydomonas reinhardtii* lacking ClpP, a plastid-encoded catalytic subunit of the Clp complex (Ramundo and Rochaix, 2014; Ramundo et al., 2014). Like the chloroplasts of *var2*, the ClpP-deficient chloroplasts of *C. reinhardtii* accumulate PQC-related proteins. Even though chloroplast proteome changes induced by the loss of Clp and FtsH2 are largely comparable, there is also an apparent difference. For instance, while chloroplast-encoded proteins remain almost unchanged in *clp*, loss of FtsH2 affects their abundances relative to wild-type plants (Zybailov et al., 2009; Dogra et al., 2019a). At present, it is unclear whether cpUPR has resulted from the general defect in chloroplast proteostasis or if the inactivation of the independent protease triggers a unique RS pathway.

Intriguingly, one *var2* allele, *var2-9*, was found to upregulate not only a suite of genes involved in PQC but also salicylic acid-responsive genes (SRGs) (Duan et al., 2019). *var2-9* mutant plants express dysfunctional FtsH2, which specifically impairs the substrate unfolding activity. This defect leads to a higher accumulation of damaged/oxidized PSII core proteins, including D1, D2, CP43, and CP47 as compared to those in *var2* null mutant plants. In addition to genes involved in PQC, *var2-9* mutant plants exhibit a significant upregulation of SRGs. It turned out that SA accumulation in *var2-9* depends on the chloroplast-established isochorismate (ICS) pathway in the absence of transcriptional upregulation of ICS-associated genes. This result suggests that the impaired proteostasis leading to the accumulation of damaged proteins may directly activate ICS enzymes to accumulate SA without transcriptional regulation. This also indicates that impaired ROS homeostasis may stimulate the ICS pathway, perhaps by altering chloroplast redox status and that the slightly accumulated SA may facilitate appropriate stress responses. Collectively, it is reasonable to propose that the ROS- (or redox)-and SA-mediated RS pathways may coordinate stress responses in response to cold, high light, and pathogen attack in which ROS and/or SA are known to accumulate.

## Either Oxidative Modification or Proteolysis Involves RS Pathways

Besides EX1, another chloroplastic oxidative stress sensor, namely 3′-phosphoadenosine 5′-phosphate (PAP) phosphatase SAL1, undergoes oxidation at specific Cysteine (Cys) residues in response to the increased level of hydrogen peroxide (H_2_O_2_) (Estavillo et al., 2011; Chan et al., 2016). In contrast to ^1^O_2_ mainly generated in PSII, H_2_O_2_ is released *via* PSI. This fact suggests that while ß-carotene and EX1 are PSII-associated ROS sensors, SAL1 is a PSI-associated ROS sensor. The Cys residue is a target of ROS since it contains a reactive thiol group, which also plays an essential role in structural modification of proteins *via* intra- and/or inter-disulfide bond formation in response to redox changes. It appears that under photo-oxidative stress conditions, specific Cys residues form inter- and intra-disulfide bonds, leading to the inactivation of SAL1 and an increase of its substrate PAP (**Figure 1C**). The PAP migrates from the chloroplasts to the nucleus to modulate the expression of a group of stress-related genes.

In contrast to EX1, Genomes Uncoupled 1 (GUN1), an integrator of multiple retrograde signals, is being stabilized under specific conditions wherein GUN1 is required to mediate RS (Wu et al., 2018; Wu et al., 2019). Given the considerable amount of *GUN1* transcripts but nearly undetectable levels of this protein, chloroplasts seem to avoid the accumulation of GUN1, perhaps because of its negative impact on chloroplast integrity under normal growth conditions (Wu et al., 2018). On the contrary, GUN1 is crucial to mediate chloroplasts-nucleus communication under certain stress conditions. As suggested for EX1, GUN1 may also facilitate RS by affecting its associated components involved in RS. Therefore, finding GUN1-associated proteins and distinguishing their molecular function seem to be essential to elucidate the mode of action of GUN1. It is important to note that it is better to reveal the GUN1 interactome under a condition where the *gun1* mutant exhibits a distinct phenotype relative to wild-type plants. This concern also can be applied for EX1 protein.

## Conclusions and Remarks

The past years of studies on chloroplast-mediated RS have unveiled different oxidative stress sensors mediating distinct RS pathways. These signaling molecules include the ^1^O_2_ sensors ß-carotene and EX1 (perhaps also EX2), and the moonlighting protein SAL1. The oxidative modifications of these sensor molecules are a prerequisite to initiating the RS pathways. Such modifications directly result from an increase in ROS levels and changes in redox status in chloroplasts. Indeed, it became apparent that chloroplasts rapidly accumulate ROS in response to various stress factors. Most likely, these sensor molecules reside close to the site of ROS production, thereby efficiently sensing the degree of stress and responding appropriately *via* RS. Even though a genuine signaling molecule acting in EX1-mediated RS remains elusive, it is rational to assume that EX1-associated proteins or EX1 per se (e.g., EX1 fragments) may release signaling molecules upon EX1 proteolysis. Besides, it is unclear: (i) how many chloroplast proteins act as ROS/redox sensors under ROS-generating stress conditions; (ii) which different stress factors activate which different ROS/redox sensor; and (iii) if ROS activate RS pathways *via* inactivation or activation of chloroplast metabolism? Resolving these questions may provide real insights for understanding the role of chloroplasts as environmental sensors in land plants.

## Author Contributions

The author confirms being the sole contributor of this work and has approved it for publication.

## Conflict of Interest

The author declares that the research was conducted in the absence of any commercial or financial relationships that could be construed as a potential conflict of interest.
